# Wengen’s hidden powers: ROS triggers a TNFR-dependent tissue regenerative pathway in *Drosophila*

**DOI:** 10.1038/s44318-024-00170-w

**Published:** 2024-07-17

**Authors:** Ditte S Andersen, Julien Colombani

**Affiliations:** Department of Biology, University of Copenhagen, Universitetsparken 15, 2100 Copenhagen, Denmark

**Keywords:** Autophagy & Cell Death, Signal Transduction

## Abstract

Recent study identifies the *Drosophila* TNF receptor Wengen as a TNF-independent mediator of tissue regeneration in response to apoptosis.

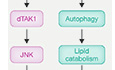

## Teaser

While the role of the Tumor necrosis factor-α (referred to as TNF here) as a key mediator of pro-inflammatory and immune responses is well-established, non-pathological functions of the TNF-TNF receptor (TNFR) signaling are less explored. In this issue of The EMBO Journal, Esteban-Collado et al, (2024) describe a TNF independent, pro-survival role of the *Drosophila* TNFR Wengen during damage-induced tissue regeneration.

Tumor necrosis factor-α (referred to as TNF here) is the founding member of a large family of TNF-related factors that regulate diverse processes ranging from cell proliferation and apoptosis to innate and adaptive immunity. Due to its implication in promoting inflammatory pathologies, such as inflammatory bowel disease (IBD), obesity-related type 2 diabetes, and cancers, the pathological properties of TNF have been particularly well studied. On the other hand, surprisingly little is known about processes regulated by the TNF-TNFR network in non-pathological conditions. This is in part due to the functional redundancy and complexity of the mammalian TNF ligand-receptor network comprising 19 ligands and 29 related TNFRs, which represents a significant obstacle in uncovering the physiological processes mediated by the TNF-TNFR network (Patel and Patel 2017). The highly simplified Drosophila TNF-TNFR system, which consists of one TNF ligand, Eiger (Egr), and two TNFRs, Grindelwald (Grnd) and Wengen (Wgn), offers a powerful alternative for studying these functions and has recently been successful in uncovering important homeostasis promoting TNF/TNFR-dependent functions (Esteban-Collado et al, 2024, Letizia et al, 2023, Loudhaief et al, 2023, Colombani and Andersen 2023, Muliyil et al, 2020).

While Wgn was identified two decades ago based on a weak homology of its cysteine-rich domains (CRD) with other members of the TNFR superfamily and its association with Egr/TNF (Kauppila et al, 2003; Kanda et al, 2002), Grnd, which shares little sequence homology with other TNFRs, was identified much later through a functional approach (Andersen et al, 2015). Recent studies have shown that Grnd and Wgn are present in different subcellular compartments, implying that these receptors fulfill distinct and non-redundant roles in cellular function. While Grnd localizes to the membrane, Wgn is primarily found in intracellular vesicles, suggesting distinct activation mechanisms for these receptors. Indeed, ectopic expression of Egr/TNF in wing imaginal disks (precursors of the adult wing) triggers the internalization of Grnd and Egr into vesicles that are separate from Wgn-positive vesicles (Palmerini et al, 2021). Consistent with this, Grnd, but not Wgn, is required for Egr-induced apoptosis (Andersen et al, 2015).

The finding that the majority of Wgn is present in intracellular vesicles, and hence not accessible for binding with extracellular Egr, raises questions about the mechanism of Wgn activation. Possible scenarios include the re-localization of Wgn to the membrane in response to specific stimuli and/or Egr-independent receptor activation. In support of the latter, Ruan et al. reported that Wgn, but not Egr, is required for the targeting of photoreceptor axons to the medulla, providing an example of a physiological process where Wgn signals independently of Egr (Ruan et al, 2013). More recently, two studies found that Wgn resides in endocytic compartments in both the gut and the trachea (the equivalent of the mammalian vasculature), where it promotes Egr/TNF-independent processes through an effect on protein stability. In the adult gut, Wgn controls dTRAF3 stability to restrict lipid catabolism and promote tissue homeostasis (Loudhaief et al, 2023), while in the trachea, it regulates receptor tyrosine kinase (RTK) stability to ensure correct specification of tracheal terminal cells (Letizia et al, 2023). These studies suggest that Wgn mediates processes that goes beyond the well-established functions of TNF/TNFRs in apoptotic, inflammatory, and immune responses and raises questions regarding how Wgn might be activated and how downstream Wgn effectors relate to the canonical dTRAF2-dTAK1-JNK signaling module employed by Grnd.

In this issue, Esteban-Collado J. et al. provide compelling evidence supporting distinct and opposing functions of Grnd and Wgn in wing imaginal disks. They demonstrate that the Grnd-dTRAF2-dTAK1-JNK signaling pathway promotes apoptosis in an Egr-dependent manner, while the Wgn-dTRAF1-Ask1-p38 MAPK module promotes survival independently of Egr. They reveal distinct responses between Grnd, which is internalized from the plasma membrane upon Egr overexpression, and Wgn, whose cytoplasmic levels decrease in Egr overexpressing cells but become enriched in neighboring wild-type cells. The non-autonomous accumulation of Wgn requires ROS, but not Egr production, by dying cells. Finally, employing an elegant binary system that allows independent gene manipulation in specific domains of the tissue, the authors establish the essential role of Wgn, but not Grnd, in the regenerative response to apoptosis, mediated through the activation and phosphorylation of p38 MAPK. These findings support a model in which ROS from damaged tissue activates Wgn-dependent signaling in surrounding cells to promote tissue regeneration. The authors speculate that ROS-dependent oxidation of cysteine residues in the CRD of Wgn may trigger self-association and recruitment of dTRAF1 to promote ligand-independent signaling.

The major significance of this work is the characterization of the distinct behaviors of the two Drosophila TNFRs, centered around their pro-apoptotic or pro-survival properties. The study nicely complements recent findings (Letizia et al, 2023; Loudhaief et al, 2023; Palmerini et al, 2021). It expands our knowledge on processes controlled by TNFR-mediated signaling, highlighting the potential for ligand-independent regulation. The understanding of the mechanistic interplay between TNFR in integrating TNF-dependent and independent signals to stimulate distinct downstream responses lays the foundation for investigating whether these insights can be generalized to other members of the TNFR superfamily. Beyond its contribution to fundamental biology, the study has biomedical implications for regenerative medicine. It emphasizes the necessity of balancing TNFR activities, downstream signaling, and their dependence on ligands, providing important insights for the development of receptor agonists or antagonists.

**Figure 1:**
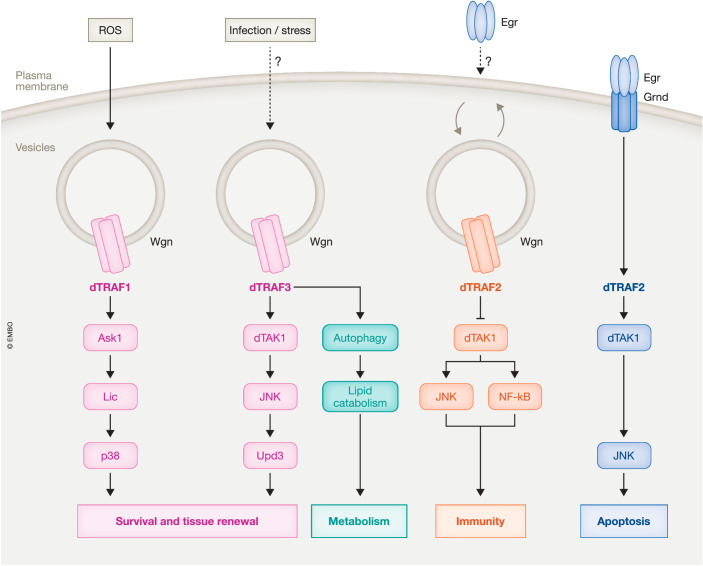
The *Drosophila* TNF/TNFR signaling pathways. The binding of the Egr/TNF to Grnd triggers an apoptotic response through the dTRAF2-JNK signaling pathway. In response to Egr-dependent and independent stimuli, Wgn associates with different dTRAFs to elicit distinct pro-survival responses.

Intriguingly, Wgn employs dTRAF2 to regulate JNK- and NF-kB-mediated effects on immunity, dTRAF3 to control lipid metabolism and tissue homeostasis, and dTRAF1 to promote Ask1-p38-mediated survival and regeneration (Fig. 1). Understanding how the activation of Wgn by different upstream signals is coupled with the recruitment of specific dTRAFs to elicit distinct cellular responses will be a fascinating direction of future research.
